# ATAD2 drives immunotherapy resistance by promoting lactic acid-mediated CD8^+^ T cell dysfunction in lung adenocarcinoma

**DOI:** 10.3389/fimmu.2026.1800533

**Published:** 2026-03-19

**Authors:** Wanfeng Gao, Jialei Xu, Yue Li, Jingchang Zhang, Chenghao Ma, Junfeng Chen, Jiajing Chen

**Affiliations:** 1 State Key Laboratory of Medicinal Chemical Biology, Institute of Immunology, College of Life Sciences, Nankai University, Tianjin, China; 2 College of Medicine, Nankai University, Tianjin, China; 3 Tianjin Nankai Hospital, Tianjin Medical University, Tianjin, China; 4 Tianjin University Central Hospital, Tianjin University, Tianjin, China; 5 Tianjin Key Laboratory of Extracorporeal Life Support for Critical Diseases, Tianjin Institute of Hepatobiliary Disease, Central Hospital, Tianjin University/Tianjin Third Central Hospital, Tianjin, China

**Keywords:** ATAD2, lactic acid, LDHA, lung adenocarcinoma, T cell therapy resistance

## Abstract

**Background:**

T cell-based immunotherapies have improved outcomes in lung adenocarcinoma (LUAD), yet many patients develop primary or acquired resistance. Tumor-intrinsic mechanisms that suppress CD8^+^ T cell function remain incompletely understood.

**Methods:**

Public LUAD transcriptomic datasets were analyzed to assess the association of ATPase family AAA domain-containing protein 2 (ATAD2) with prognosis and immune infiltration. *Atad2*-deficient LUAD cell lines were generated using CRISPR/Cas9 and co-cultured with activated CD8^+^ T cells to evaluate cytotoxicity, cytokine production, PD-1 expression and survival. The mediating role of lactic acid (LA) was confirmed using conditioned medium exposure, exogenous LA supplementation, and LDHA overexpression rescue experiments. ATAD2-mediated transcriptional regulation of LDHA was investigated by ChIP-qPCR and c-Myc overexpression. Subcutaneous tumor models were used to determine the effects of *Atad2* deletion on LA accumulation, CD8^+^ T cell infiltration, tumor growth, and response to anti-PD-1 therapy.

**Results:**

ATAD2 was significantly upregulated in LUAD and correlated with poor survival and decreased CD8^+^ T cell infiltration. *Atad2* deletion enhanced CD8^+^ T cell function and survival, effects reversed by exogenous LA. Mechanistically, ATAD2 enhanced c-Myc-dependent LDHA transcription, leading to increased lactic acid production and an immunosuppressive microenvironment. LDHA overexpression restored LA levels and reversed the immune-activating effects of *Atad2* loss. *In vivo*, *Atad2* deficiency reduced intratumoral LA, remodeled the immunosuppressive microenvironment, increased CD8^+^ T cell infiltration, inhibited tumor growth, and improved sensitivity to anti-PD-1 therapy.

**Conclusions:**

ATAD2 drives immunotherapy resistance in LUAD by activating an ATAD2-LDHA-LA axis that impairs CD8^+^ T cell function. Targeting ATAD2 may broadly restore antitumor immunity and enhance the efficacy of T cell-based immunotherapies.

## Introduction

Lung adenocarcinoma (LUAD), the most prevalent subtype of non-small-cell lung cancer (NSCLC), remains a leading cause of cancer-related mortality worldwide despite advances in targeted and immunotherapies (1). The advent of T cell-dependent immunotherapies, particularly immune checkpoint inhibitors (ICIs), has revolutionized LUAD treatment by re-activating exhausted CD8^+^ T cells. However, only a subset of patients achieved durable clinical benefit (2). This highlights an urgent need to uncover tumor-intrinsic mechanisms that confer resistance to T cell-mediated immune surveillance.

LUAD cells employ multiple intrinsic mechanisms to suppress CD8^+^ T cell function. Genetically, mutations in chromatin remodelers such as SMARCA4 disrupt the expression of immune-related genes, impairing T cell priming (3). Epigenetically, overexpression of CK2B drives T cell exhaustion via HDAC8-mediated reprogramming (4). Metabolically, the intrinsic Warburg effect of LUAD cells leads to lactic acid (LA) accumulation, thereby suppressing CD8^+^ T cell cytotoxicity and promoting exhaustion (5, 6). Additionally, tumor cells upregulate PD-L1 via GOLM1 to directly inhibit T cell function, and oncogenic EGFR mutations result in reduced T cell infiltration (7, 8). Collectively, these multifaceted intrinsic alterations not only hinder CD8^+^ T cell activity but also limit the efficacy of immunotherapies. Among these, metabolic reprogramming-particularly LA accumulation-has emerged as a central node integrating oncogenic signals and immune suppression. However, the upstream epigenetic regulators that orchestrate this metabolic-immune crosstalk remain poorly defined.

ATPase family AAA domain-containing protein 2 (ATAD2) is an epigenetic co-activator characterized by an AAA^+^ ATPase domain and a bromodomain that binds acetylated histones to promote oncogenic transcriptional programs (9). ATAD2 is overexpressed in multiple human malignancies-including breast, gastric cancer, hepatocellular, and ovarian cancer-and its upregulation correlates with aggressive clinical behavior and poor prognosis (10–13). In LUAD, ATAD2 promotes proliferation and epithelial-mesenchymal transition via the PI3K/AKT axes (14–16). However, whether ATAD2 contributes to immune evasion or resistance to T cell-dependent immunotherapy in LUAD remains unknown. Deciphering this link could reveal a critical epigenetic-metabolic axis driving immunotherapy failure.

In this study, we investigated whether ATAD2 drives T cell therapy resistance in LUAD through metabolic reprogramming. Using transcriptomic analyses of public LUAD datasets, we first confirmed that ATAD2 is markedly upregulated and correlates with poor survival and reduced CD8^+^ T cell infiltration. Through *in vitro* co-culture assays, we demonstrate that *Atad2* deletion enhances CD8^+^ T cell-mediated cytotoxicity and cytokine production, effects that are reversed by exogenous LA supplementation. Mechanistically, ATAD2 enhances the expression of LDHA via c-Myc-dependent mechanism, driving LA production and creating an immunosuppressive tumor microenvironment (TME). Finally, *in vivo* models validate that *Atad2* knockout decreases intratumoral LA and remodels the immunosuppressive tumor microenvironment. Specifically, it enhances CD8^+^ T cell, dendritic cell, and NK cell infiltration while reducing Tregs, MDSCs, and M2-like TAMs. Consequently, *Atad2* deficiency suppresses tumor growth and sensitizes tumors to anti-PD-1 treatment. Collectively, these findings establish ATAD2 as a potential master regulator linking metabolism and T cell immunotherapy evasion in LUAD.

## Materials and methods

### Database analyses

The expression patterns and clinical relevance of ATAD2 and LDHA were analyzed using publicly available databases. Expression profiles of ATAD2 and LDHA were retrieved from the GEPIA database (http://gepia2021.cancer-pku.cn/). Protein expression patterns of ATAD2 were further examined using the Human Protein Atlas (HPA, https://www.proteinatlas.org/). The Kaplan-Meier Plotter (https://kmplot.com/analysis/) was applied to assess the prognostic significance of ATAD2 and LDHA. Correlation analyses and immune infiltration evaluations of these genes were conducted through the TIMER platform (http://timer.cistrome.org/).

### Cell culture

The mouse lung cancer cell lines LLC and CMT167 were purchased from ATCC. They were cultured in DMEM supplemented with 10% FBS and 1% penicillin-streptomycin and maintained at 37 °C in a 5% CO_2_ humidified incubator. Routine mycoplasma testing was performed to ensure cell authenticity.

### Plasmid transfection

To establish *Atad2*-deficient cell lines, two sgRNAs targeting the *Atad2* coding region (sgRNA-1: ACCACAACACGCGCTCTTTA; sgRNA-2: TTCCTCAATGAGCGAATAAC) were inserted into the pSpCas9(BB)-2A-GFP backbone (Addgene 48138). LLC and CMT167 cells were co-transfected with both constructs using Lipofectamine 3000 (Invitrogen, L3000015), while cells receiving the empty vector served as controls. Gene knockout efficiency was confirmed by Western blotting. For overexpression assays, full-length ATAD2, LDHA or c-Myc cDNAs were subcloned into the pcDNA3.1 expression vector and delivered into cells with Lipofectamine 3000 in accordance with the manufacturer’s instructions.

### Western blotting assay

Total proteins were extracted in RIPA lysis buffer containing protease and phosphatase inhibitors. Equal amounts of protein were separated by SDS-PAGE and transferred onto PVDF membranes. Following blocking with 5% non-fat milk, the membranes were incubated overnight at 4 °C with primary antibodies against ATAD2 (Cell signaling Technology, 50563), LDHA (Cell signaling Technology, 2012), or β-actin (PTM Biolabs, PTM-5028), and then with HRP-linked secondary antibodies. Protein signals were visualized using enhanced chemiluminescence (ECL) reagents.

### RNA extraction and qRT-PCR

Total RNA was isolated from cultured cells using the RNAfast200 kit (Fastagen, 220011) according to the manufacturer’s protocol. First-strand cDNA was synthesized from the extracted RNA using the RT Master Mix (TOYOBO, FSQ-201). Quantitative real-time PCR was carried out with SYBR^®^ qPCR Master Mix (TOYOBO, QPK-201) on a QuantStudio 7 Flex Real-Time PCR System (Thermo Fisher Scientific). Gene expression was quantified using the comparative Ct method and normalized to *β-actin* expression. The primer sequences used for qPCR were as follows:

*Ldha*: Forward, 5′-AACTTGGCGCTCTACTTGCT-3′; Reverse, 5′-GGACTTTGAATCTTTTGAGACCTTG -3′.
*β-actin*: Forward, 5′-CGCAGCCACTGTCGAGTC-3′; Reverse, 5′-GTCATCCATGGCGAACTGGT-3′.

### Chromatin immunoprecipitation-qPCR

Chromatin immunoprecipitation followed by qPCR (ChIP-qPCR) was performed using ChIP kit (Cell Signaling Technology, 9005) according to the manufacturer’ instructions. Briefly, cells were fixed in 1% formaldehyde to stabilize protein-DNA interactions and then lysed. The chromatin was sheared by sonication to obtain fragments of approximately 200-1,000 bp. The diluted lysates were incubated overnight at 4 °C with c-Myc antibody (Cell Signaling Technology, 9402), while normal IgG served as the negative control. Immunocomplexes were captured, washed, and eluted, and the cross-links were reversed before DNA purification. Enrichment of the LDHA promoter region was quantified by SYBR-based real-time PCR. Primer sequences used for ChIP-qPCR were as follows: Forward, 5’-CCTTCTTTGGGGTGTCGCAGCA-3’; Reverse, 5’-CCAGCGGACGTGCGGGAACC-3’.

### Co-culture of effector-to-target cells

Naïve CD8^+^ T cells were isolated from mouse spleens using the EasySep™ Mouse Naïve CD8^+^ T Cell Isolation Kit (STEMCELL Technologies, 19858). Purified cells were activated with plate-bound anti-CD3 (10 µg/mL) and soluble anti-CD28 (5 µg/mL) in RPMI 1640 medium supplemented with 10% FBS, 1% penicillin-streptomycin, 50 µM β-mercaptoethanol, and 1% GlutaMAX. After 48 h of stimulation, activated CD8^+^ T cells were co-cultured with indicated LLC or CMT167 cells for 6 hours at effector-to-target ratios of 1:5 and 1:10, respectively. Cytotoxicity was assessed using the CytoTox 96 Non-Radioactive Cytotoxicity Assay (Promega, G1780).

### Preparation of the conditioned medium

LLC and CMT167 cells transduced with the indicated plasmids were seeded at 2×10^6^ cells per 10-cm dish and allowed to adhere overnight. The cells were then washed twice with PBS and the culture medium was replaced with fresh DMEM medium supplemented with 1% FBS. After 24 hours incubation, the supernatant was collected and centrifuged at 3,000 g for 10 minutes to remove residual debris. The clarified supernatant was subsequently passed through a 0.22 μm filter and designated as conditioned medium (CM). Aliquots were stored at -80 °C until use.

### ELISA assay

Activated CD8^+^ T cells were cultured in indicated CM for 6 hours. The concentrations of IFN-γ (Abcam, ab282874), TNF-α (Abcam, ab208348), and Granzyme B (Abcam, ab238265) in the culture supernatants were determined using ELISA kits according to the manufacturer’s instructions.

### Surface PD−1 analysis

Following activation, CD8^+^ T cells were incubated with CM from the indicated LLC or CMT167 cells for 6 hours. And then, cells were stained with APC anti-PD-1 (Biolegend, 135210) for 30 minutes at 4 °C in the dark, and PD-1 surface expression was analyzed using FACS (BD, LSRFortessa™ X-20).

### Cell viability assay

Activated CD8^+^ T cells were exposed to the indicated CM for 24 hours. Cell viability was determined using the Calcein/PI Cell Viability/Cytotoxicity Assay Kit (Beyotime, C2015M), according to the manufacturer’s instructions.

### Lactate analysis

Lactate concentrations in the culture supernatants of indicated LLC or CMT167 cells, as well as in their corresponding tumor tissues, were measured with a Lactate Assay Kit (Abcam, ab65331) according to the manufacturer’s protocol.

### *In vivo* animal studies

C57BL/6 mice were purchased from Beijing Vital River Laboratory Animal Technology Co., Ltd and maintained under specific pathogen-free (SPF) conditions at Nankai University. All procedures were performed with approval from the Nankai University Laboratory Animal Welfare Ethics Committee (No. 2021-SYDWLL-000355). To establish tumors, 5 × 10^5^ indicated LLC or CMT167 cells suspended in 100 μL PBS were subcutaneously implanted into the right flank. For anti-PD-1 treatment, 200 μg of anti-PD-1 (BioXCell, BE0273) or Isotype control IgG2a (BioXCell, BE0089) was injected intraperitoneally every three days, commencing when tumor volumes reached ~200 mm^3^. Tumor growth was monitored every three days, and tumor volume was calculated using the formula: length × width^2^/2. Mice were euthanized once tumors reached approximately 2000 mm³ by CO2 asphyxiation at a flow rate of 30% of the chamber volume per minute, followed by cervical dislocation.

### Flow cytometry

Primary tumors from tumor-bearing mice were collected and processed as previously reported (17, 18). In brief, tumors were mechanically minced and digested at 37°C for 60 minutes in a solution containing 0.3 mg/mL collagenase type I (Gibco, 17018029), 1 mg/mL collagenase type IV (Gibco, 17104019), and 1 mg/mL DNase I (Sigma-Aldrich, DN25). The digests were passed through a 40 μm cell strainer to obtain single-cell suspensions, followed by removal of erythrocytes using erythrocyte lysis buffer (Invitrogen, 00-4333-57). Cells were first treated with TruStain FcX (BioLegend, 101320) for 10 minutes at 4°C to block Fc-mediated non-specific binding. They were then stained in the dark at 4°C for 30 minutes with Zombie-Aqua (BioLegend, 423102), APC/Cyanine7 anti-CD45 (BioLegend, 103116), PerCP/Cyanine5.5 anti-CD3 (BioLegend, 100218), Brilliant Violet 605TM anti-CD8 (BioLegend, 100744), PE/Cyanine7 anti-NK1.1 (BioLegend, 156514), APC anti-CD3 (BioLegend, 100236), FITC anti-CD4 (BioLegend, 100406), PerCP/Cyanine5.5 anti-CD25 (BioLegend, 101911), PE anti-Foxp3 (BioLegend, 126403), FITC anti-Ly-6G/Ly-6C (Gr-1) (BioLegend, 108405), PE anti-CD11b (BioLegend, 101208), Percp/cy5.5 anti-F4/80 (BioLegend, 123127), FITC anti- CD206 (MMR) (BioLegend, 141703), Brilliant Violet 650™ anti-CD45 (BioLegend, 103151), PE/Cyanine7 anti-CD11c (BioLegend, 117318), and APC/Cyanine7 anti-I-A/I-E (BioLegend, 107628).

### Tumor tissue immunofluorescence, TUNEL staining, and immunohistochemistry

Tumor tissues were snap-frozen, embedded in O.C.T., and cryosectioned. For immunofluorescence staining, sections were fixed in 4% PFA, blocked with 5% BSA, and incubated overnight at 4 °C with Alexa Fluor 647-anti-CD8a (BioLegend, 100727). Slides were mounted with antifade medium (Beyotime, P0126) and imaged by confocal microscopy (A1+, Nikon). Apoptotic cells were detected using a TUNEL assay kit (Beyotime, C1088) following the manufacturer’s protocol. For immunohistochemistry, frozen sections underwent citrate-based antigen retrieval, serum blocking, and overnight incubation with LDHA antibody at 4 °C. After incubation with secondary antibody, staining was visualized using a DAB kit (Dako, K5007), followed by counterstaining, dehydration, and mounting.

### Statistical analysis

All statistical analyses were carried out using GraphPad Prism software. Differences between groups were assessed with a two-tailed unpaired Student’s t-test, one-way ANOVA, or the log-rank test, as appropriate. A P value < 0.05 was considered statistically significant (*P < 0.05; **P < 0.01; ***P < 0.001; ****P < 0.0001).

## Results

### ATAD2 is upregulated in LUAD and associated with poor prognosis and impaired CD8^+^ T cell infiltration

**Figure 1:**
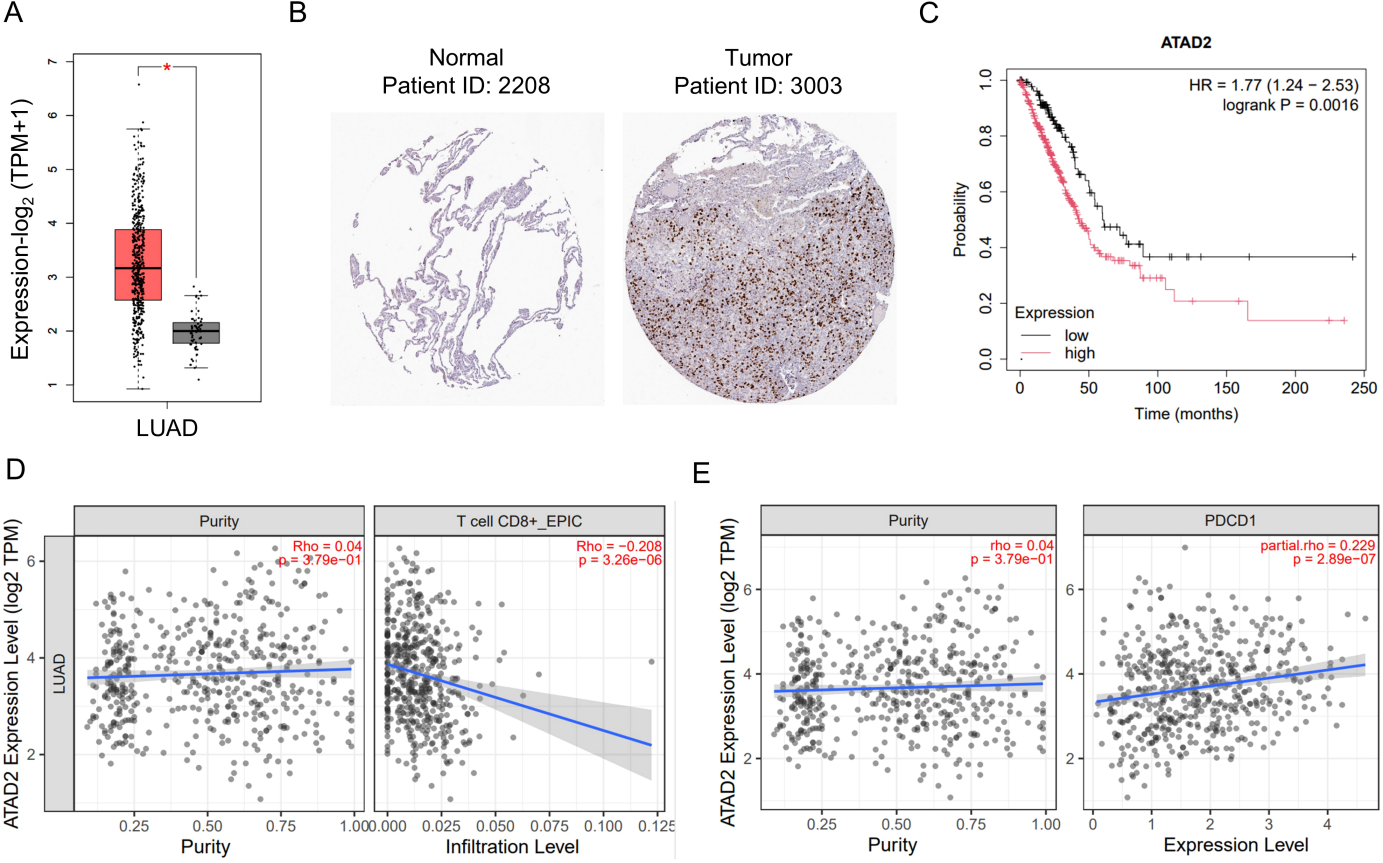
Elevated ATAD2 expression in LUAD and its association with reduced CD8^+^ T cell infiltration. **(A)** ATAD2 expression levels in in lung adenocarcinoma (LUAD) and normal samples from the GEPIA database. **(B)** Immunohistochemical staining of ATAD2 in LUAD (patient ID: 3003) and normal tissue (patient ID: 2208) samples from the Human Protein Atlas. **(C)** Kaplan-Meier survival analysis comparing LUAD patients with high versus low ATAD2 expression, showing the correlation between ATAD2 levels and patient survival. **(D)** Correlation analysis of ATAD2 expression with CD8^+^ T cell infiltration levels in LUAD, as predicted by the TIMER database. **(E)** The relationship between ATAD2 and PDCD1 expression levels in LUAD samples as predicted by TIMER.

To determine the clinical relevance of ATAD2 in lung adenocarcinoma (LUAD), we first analyzed its expression profile in public datasets. ATAD2 expression was markedly elevated in LUAD tissues compared with normal lung samples (**Figures 1A, B**). Kaplan-Meier analysis further revealed that patients with higher ATAD2 expression exhibited significantly poorer overall survival (**Figure 1C**). To assess whether ATAD2 is associated with the T cell therapy resistance, we next examined its relationship with CD8^+^ T cell infiltration. ATAD2 expression exhibited an inverse association with CD8^+^ T cell infiltration and a positive correlation with the exhaustion marker PDCD1. These findings indicate its potential involvement in driving T cell dysfunction within the tumor microenvironment (**Figures 1D, E**). Collectively, these results indicate that ATAD2 is upregulated in LUAD and is linked to both adverse prognosis and altered CD8^+^ T cell infiltration patterns.

### ATAD2 inhibits CD8^+^ T cell-mediated antitumor immunity

**Figure 2:**
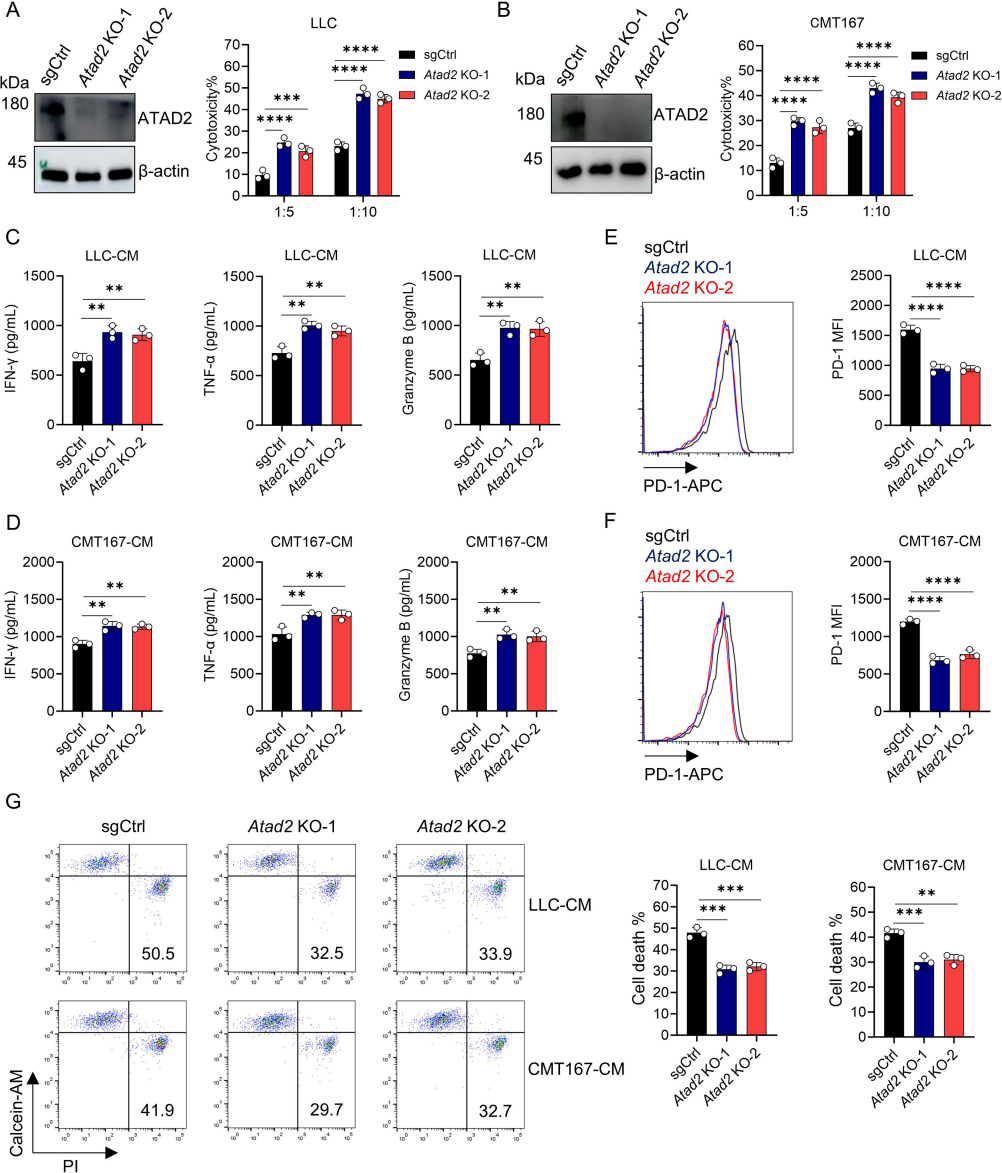
*Atad2* knockout enhances CD8^+^ T cell-mediated antitumor immunity. **(A)** Western blot analysis of ATAD2 and β-actin expression in LLC cells after transduced with vector or *Atad2* knockout (KO) plasmid (left), and the cytotoxic activity of activated CD8^+^ T cells after co-culture with these cells (right). **(B)** Western blot showing ATAD2 and β-actin expression in CMT167 cells expressing vector or *Atad2* KO plasmid (left), together with the cytotoxic response of activated CD8^+^ T cells following co-culture (right). **(C, D)** Expression levels of IFN-γ, TNF-α, and Granzyme B in activated CD8^+^ T cells cultured with conditioned medium (CM) derived from LLC **(C)** or CMT167 cells **(D)** expressing the indicated plasmids. (n=3). **(E, F)** Surface PD-1 expression in activated CD8^+^ T cells exposed to CM collected from LLC **(E)** or CMT167 cells **(F)** transduced with the indicated plasmids. (n=3). **(G)** Cell death of activated CD8^+^ T cells exposed to CM collected from LLC or CMT167 cells transduced with the indicated plasmids. (n=3). Data are presented as mean ± SD of biological replicates. Representative results from one of three independent experiments are shown. **P < 0.01; ***P < 0.001; ****P < 0.0001.

To further elucidate the functional role of ATAD2 in T cell-mediated immunity, we established a co-culture model of activated CD8^+^ T cells and LUAD cells. Genetic ablation of *Atad2* markedly enhanced the cytolytic activity of CD8^+^ T cells, as evidenced by a significant increase in target-cell lysis (**Figures 2A, B**). To investigate the mechanism by which *Atad2* deficiency enhances CD8^+^ T cell function in the co-culture system, we collected CM from LUAD cells with or without *Atad2* knockout. Functional analyses demonstrated that CM derived from *Atad2*-deficient LUAD cells markedly enhanced CD8^+^ T cell activity. This was reflected by increased secretion of effector cytokines, including IFN-γ, TNF-α, and granzyme B (**Figures 2C, D**). Moreover, exposure to this CM markedly diminished PD-1 expression on CD8^+^ T cells (**Figures 2E, F**) and significantly curtailed CD8^+^ T cell death (**Figure 2G**). Collectively, our data suggest that ATAD2 acts as a negative regulator of antitumor immunity, linking its oncogenic upregulation in LUAD with T cell immunotherapy evasion mechanisms.

### ATAD2 drives CD8^+^ T cell dysfunction by regulating lactic acid production

**Figure 3:**
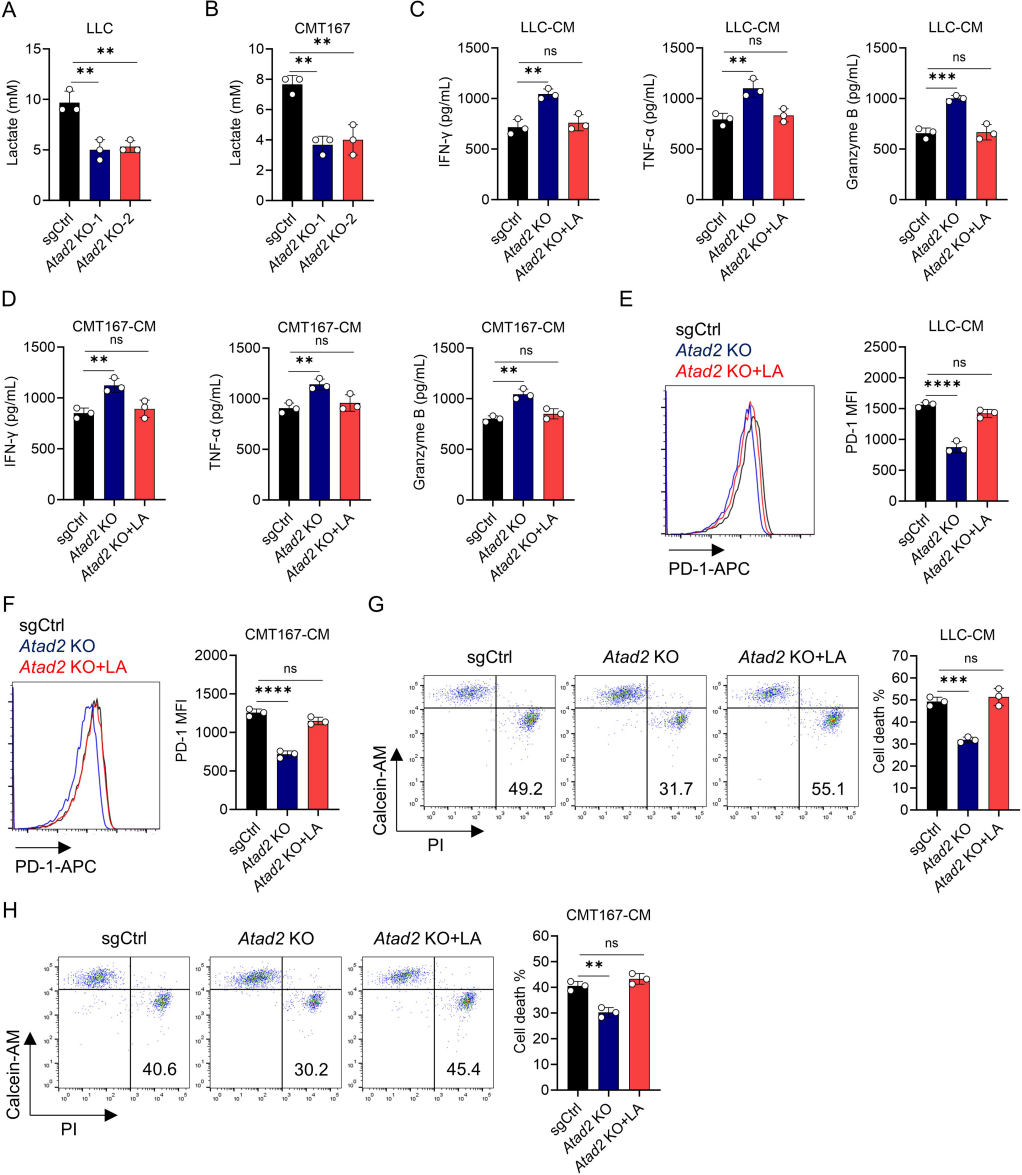
ATAD2 enhances lactic acid production and promotes CD8^+^ T cell dysfunction. **(A, B)** Measurement of lactate concentration in the conditioned medium (CM) from LLC **(A)** or CMT167 cells **(B)** transduced with the indicated plasmids. (n=3). **(C, D)** Production of IFN-γ, TNF-α, and Granzyme B in activated CD8^+^ T cells after incubation with CM from indicated LLC **(C)** or CMT167 cells **(D)** in the presence or absence of 5 mM LA supplementation. (n=3). **(E, F)** PD-1 expression in activated CD8^+^ T cells after incubation with CM from indicated LLC **(E)** or CMT167 cells **(F)** with or without 5 mM LA supplementation. (n=3). **(G, H)** Cell death of activated CD8^+^ T cells after incubation with CM from indicated LLC **(G)** or CMT167 cells **(H)** with or without 5 mM LA supplementation. (n=3). Data are presented as mean ± SD of biological replicates. Representative results from one of three independent experiments are shown. **P < 0.01; ***P < 0.001; ****P < 0.0001; ns, not significant.

Based on the well-documented immunosuppressive function of LA, we sought to investigate whether it contributes to ATAD2-dependent T cell therapy resistance (19). Evaluation of LA secretion revealed that *Atad2* knockout significantly suppressed its production in LUAD cells (**Figures 3A, B**). To further confirm that LA serves as the key mediator in this process, we conducted a series of functional rescue experiments. Upon addition of exogenous LA to the CM obtained from *Atad2*-deficient LUAD cells, the immune-enhancing effects were effectively abrogated. In this setting, CD8^+^ T cells displayed substantially diminished production of IFN-γ, TNF-α, and granzyme B, together with elevated PD-1 expression and increased rates of cell death (**Figures 3C–H**). Together, these findings strongly indicate that ATAD2 promotes immune escape in LUAD by elevating tumor-derived LA levels, thereby functionally suppressing CD8^+^ T cell responses and facilitating resistance to T cell-based immunotherapy.

### ATAD2 upregulates LDHA expression through enhancing c-Myc-dependent transcription

**Figure 4:**
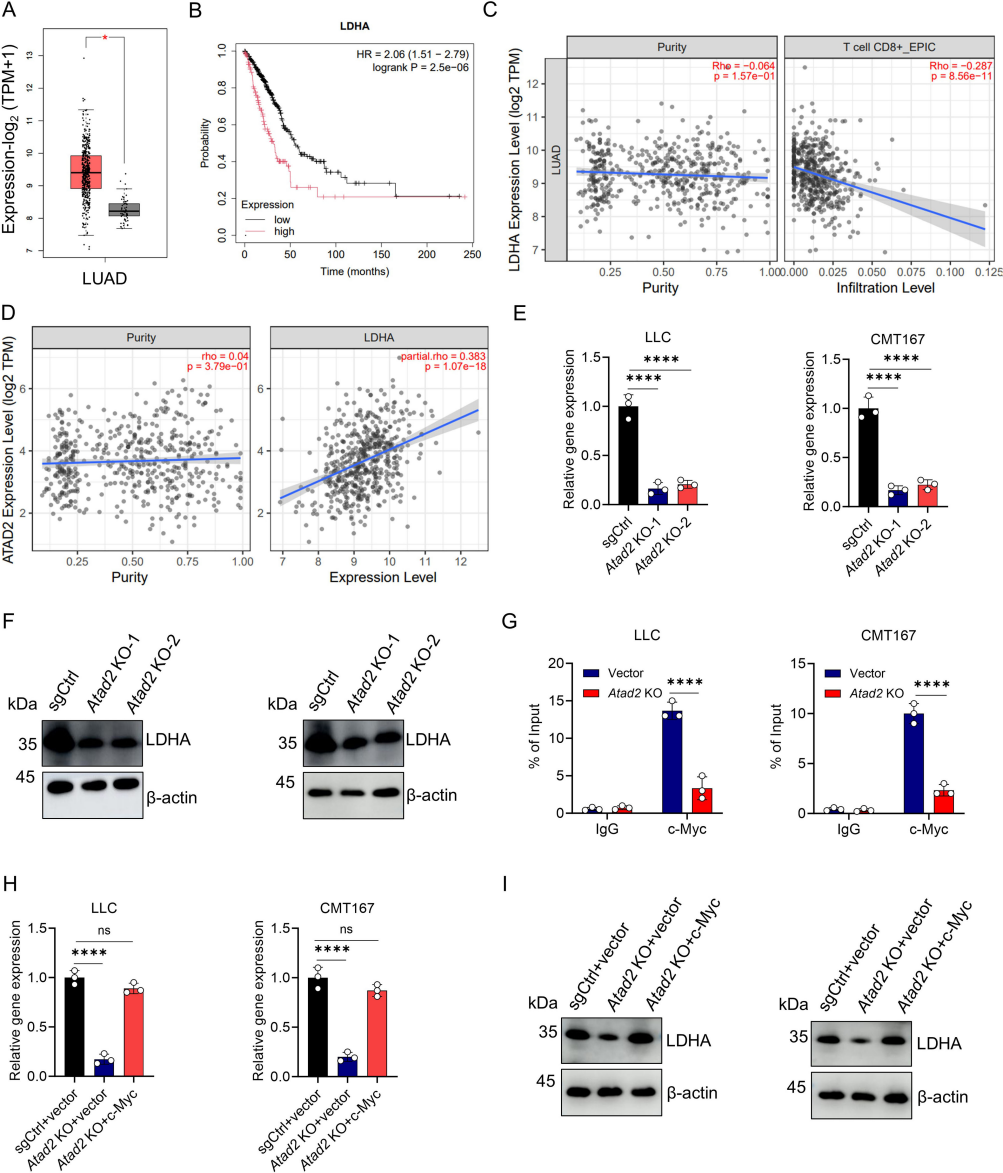
ATAD2 positively correlates with LDHA expression and regulates its transcriptional and protein levels. **(A)** Comparative analysis of LDHA expression between LUAD and normal tissues using the GEPIA database. **(B)** Kaplan-Meier survival curves of LUAD patients showing poorer prognosis associated with elevated LDHA expression. **(C)** Association between LDHA expression and CD8^+^ T cell infiltration predicted by TIMER. **(D)** Correlation analysis between ATAD2 and LDHA expression levels in lung adenocarcinoma (LUAD) samples using TIMER. **(E)** Relative LDHA gene expression analysis of LLC (left) and CMT167 (right) cells after *Atad2* knockout. (n=3). **(F)** Western blot detection of LDHA and β-actin protein levels in LLC (left) and CMT167 (right) cells following *Atad2* knockout. **(G)** ChIP-PCR analysis of c-Myc binding to the LDHA locus in LLC (left) and CMT167 (right) cells. IgG was used as a negative control. (n=3). **(H)** Relative LDHA mRNA levels of LLC (left) and CMT167 (right) after transfected with indicated plasmids. (n=3). **(I)** Western blot detection of LDHA and β-actin protein levels in indicated LLC (left) and CMT167 (right) cells. Data are presented as mean ± SD of biological replicates. Representative results from one of three independent experiments are shown. *P < 0.05. **P < 0.01; ***P < 0.001; ****P < 0.0001; ns, not significant.

LDHA is a key LA-producing enzyme that is upregulated in LUAD and correlates with poor prognosis and reduced CD8^+^ T cell infiltration (**Figures 4A–C**). Its expression also strongly associates with ATAD2 (**Figure 4D**). Therefore, we hypothesized that ATAD2 regulates LA levels by modulating LDHA expression. To test this hypothesis, we evaluated LDHA expression following *Atad2* deletion in mouse LUAD cell lines. *Atad2* knockout significantly reduced LDHA mRNA abundance in both LLC and CMT167 cells (**Figure 4E**), which was accompanied by a marked decrease in LDHA protein levels (**Figure 4F**). ATAD2 has been reported as a transcriptional co-activator of c-Myc, and LDHA is a well-established c-Myc target gene (20–24). Therefore, we next examined whether ATAD2 modulates LDHA through c-Myc-dependent transcription. ChIP-qPCR experiments confirmed robust binding of c-Myc to the LDHA promoter in control cells, whereas this enrichment was dramatically reduced upon *Atad2* knockout (**Figure 4G**). Consistently, forced expression of c-Myc restored LDHA mRNA and protein levels that were otherwise suppressed by *Atad2* deletion in both LLC and CMT167 cells (**Figures 4H, I**). Collectively, these data demonstrate that ATAD2 positively regulates LDHA expression by facilitating c-Myc recruitment to the LDHA promoter. This links ATAD2 activity to enhanced LA-producing metabolism and immune suppression in LUAD.

### ATAD2 impairs CD8^+^ T cell function through LDHA-dependent lactic acid production

**Figure 5:**
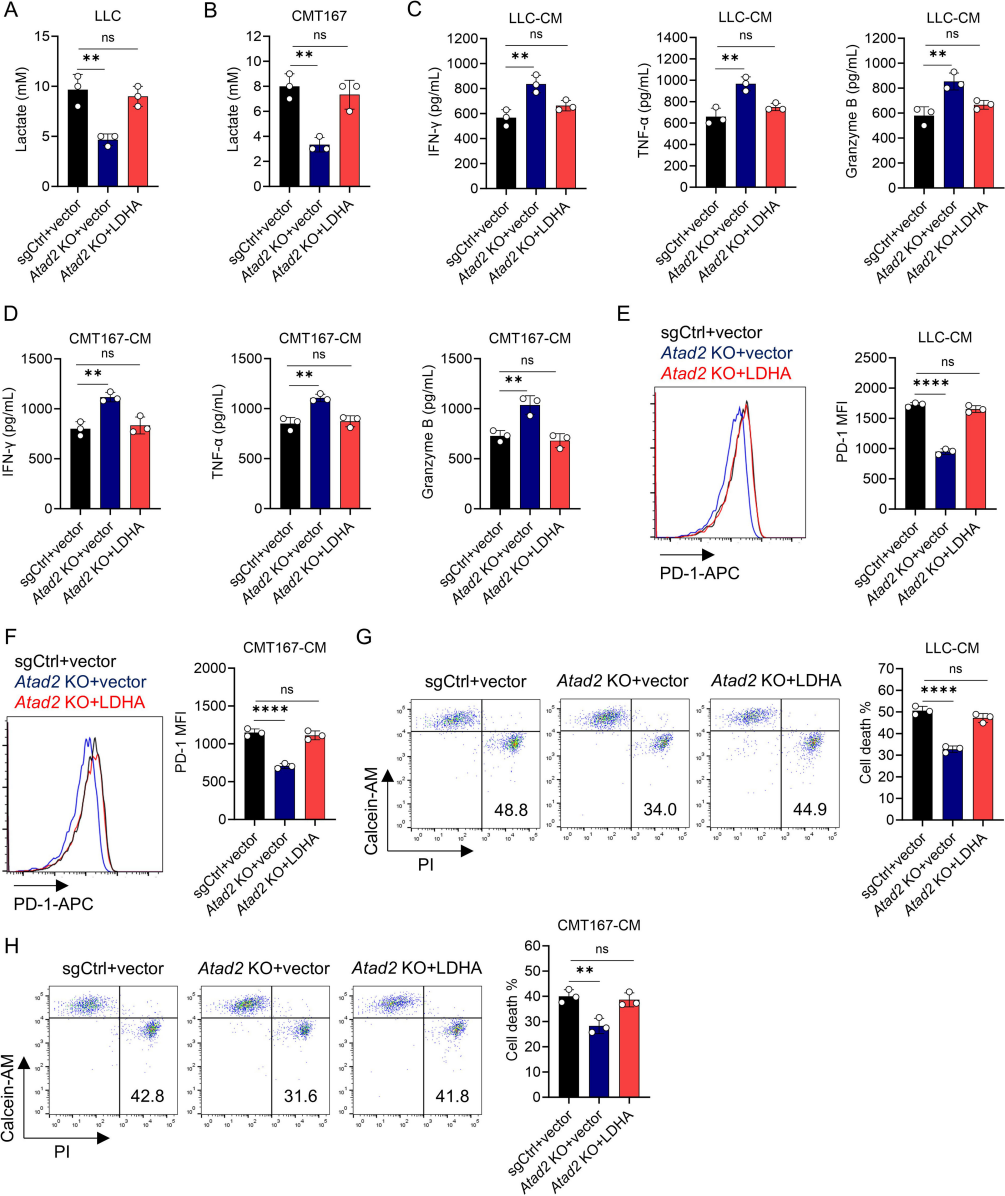
LDHA overexpression reverses the immunostimulatory effects of *Atad2* deficiency. **(A, B)** Quantification of lactate concentrations in the conditioned medium (CM) collected from the LLC **(A)** or CMT167 cells **(B)** expressing the indicated plasmids. (n=3). **(C, D)** Levels of IFN-γ, TNF-α, and Granzyme B secreted by CD8^+^ T cells exposed to CM derived from the LLC **(C)** or CMT167 cells **(D)** transduced with the indicated plasmids. (n=3). **(E, F)** Surface PD-1 expression in activated CD8^+^ T cells after incubation with CM obtained from the indicated LLC **(E)** or CMT167 cells **(F)**. (n=3). **(G, H)** Cell death of activated CD8^+^ T cells after incubation with CM from indicated LLC **(G)** or CMT167 cells **(H)**. (n=3). Data are presented as mean ± SD of biological replicates. Representative results from one of three independent experiments are shown. **P < 0.01; ****P < 0.0001; ns, not significant.

To definitively establish LDHA as the critical downstream effector in the ATAD2 signaling axis, we conducted a series of genetic rescue experiments. Ectopic overexpression of LDHA in *Atad2*-deficient LUAD cells effectively restored LA production, which was initially suppressed by the knockout (**Figures 5A, B**). CM derived from these LDHA-overexpressing, *Atad2*-deficient cells re-established a distinctly immunosuppressive milieu, leading to a pronounced impairment of CD8^+^ T cell effector activity. This was reflected by diminished secretion of IFN-γ, TNF-α, and granzyme B (**Figures 5C, D**), together with a parallel increase in PD-1 expression and a higher incidence of cell death (**Figures 5E–H**). Collectively, these rescue experiments provide compelling functional evidence that LDHA operates as the key mediator through which ATAD2 suppresses antitumor immunity. Taken together with our earlier observations, these data delineate an ATAD2-LDHA-LA metabolic axis. This axis fosters an immunosuppressive tumor microenvironment and ultimately contributes to immune evasion in LUAD.

### *Atad2* deficiency inhibits lactic acid production and tumor growth *in vivo*

**Figure 6:**
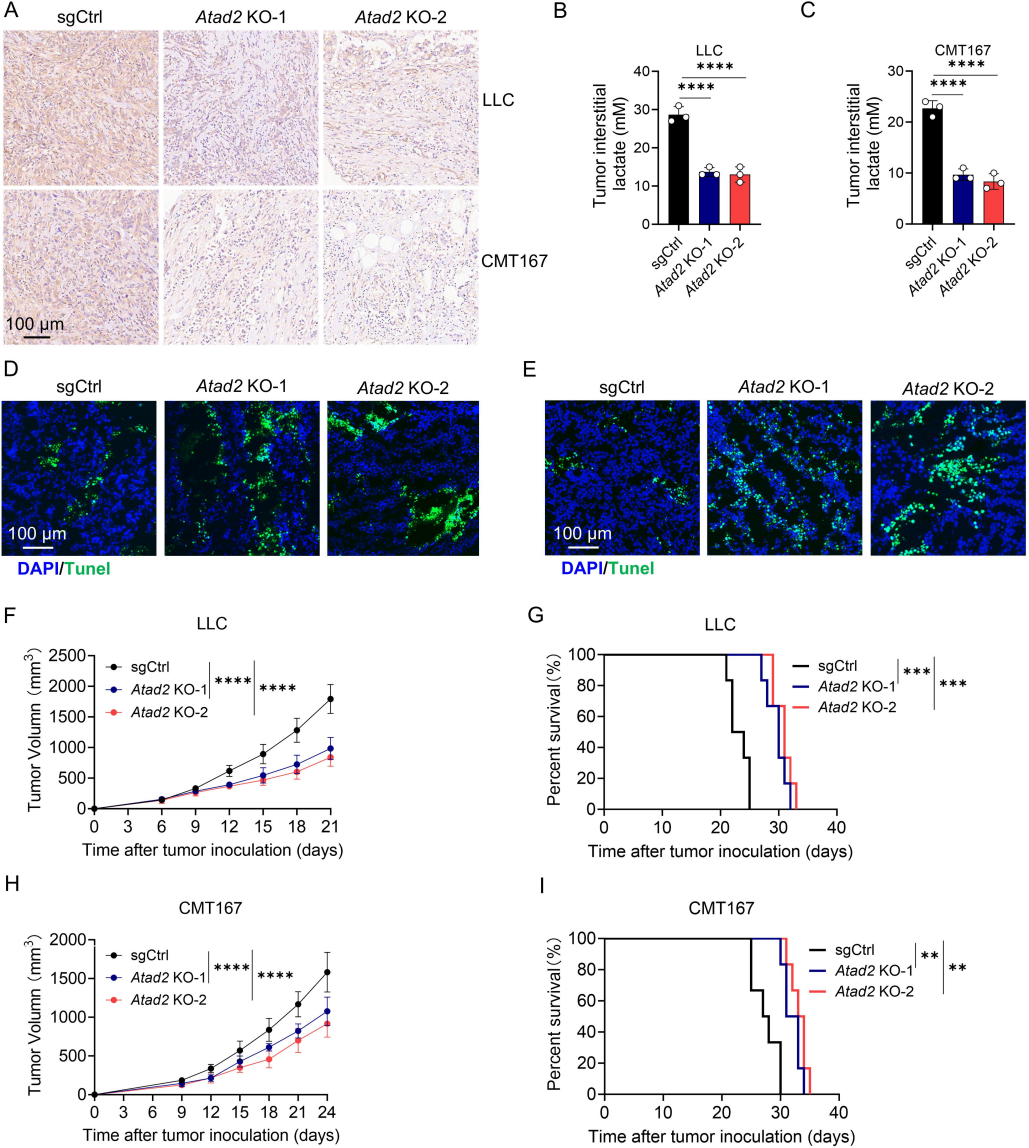
*Atad2* knockout suppresses lactic acid accumulation and tumor progression *in vivo*. **(A)** Immunohistochemical staining for LDHA expression in indicated LLC or CMT167 tumor tissues. (n=3). **(B, C)** Measurement of lactate concentrations within the indicated LLC **(B)** or CMT167 tumors **(C)**. (n=3). **(D, E)** TUNEL staining of indicated LLC **(D)** or CMT167 **(E)** tumors. (n=3). **(F, G)** Tumor growth kinetics **(F)** and overall survival **(G)** of mice implanted with LLC cells expressing the indicated plasmids. (n=6). **(H, I)** Tumor volume progression **(H)** and survival curves **(I)** of mice bearing CMT167 tumors stably transduced with the indicated plasmids. (n=6). Data are presented as mean ± SD of biological replicates. Representative results from one of three independent experiments are shown. **P < 0.01; ***P < 0.001; ****P < 0.0001.

To determine the functional relevance of ATAD2 in regulating tumor metabolism and growth *in vivo*, we established subcutaneous LLC and CMT167 tumor models using control or *Atad2*-deficient cells. Immunohistochemical analysis demonstrated a marked reduction in LDHA expression in *Atad2*-knockout tumors compared with the control group (**Figure 6A**). Consistent with this finding, intratumoral lactate concentrations were substantially decreased in both LLC and CMT167 tumors lacking *Atad2*, indicating that *Atad2* loss impairs tumor-associated LA accumulation (**Figures 6B, C**). We next examined the impact of *Atad2* deletion on tumor cell viability *in vivo*. TUNEL staining revealed a pronounced increase in apoptotic cells within *Atad2*-deficient tumors relative to controls in both tumor models (**Figures 6D, E**). Functionally, *Atad2* knockout significantly delayed tumor growth in mice implanted with either LLC or CMT167 cells (**Figures 6F, H**). Moreover, animals bearing *Atad2*-deficient tumors exhibited markedly prolonged overall survival compared with control tumor-bearing mice (**Figures 6G, I**). Collectively, these data demonstrate that genetic abolition of *Atad2* suppresses LDHA expression and LA production in the tumor microenvironment. This restrains tumor progression and extends host survival *in vivo*.

### *Atad2* deficiency remodels the tumor immune landscape

**Figure 7:**
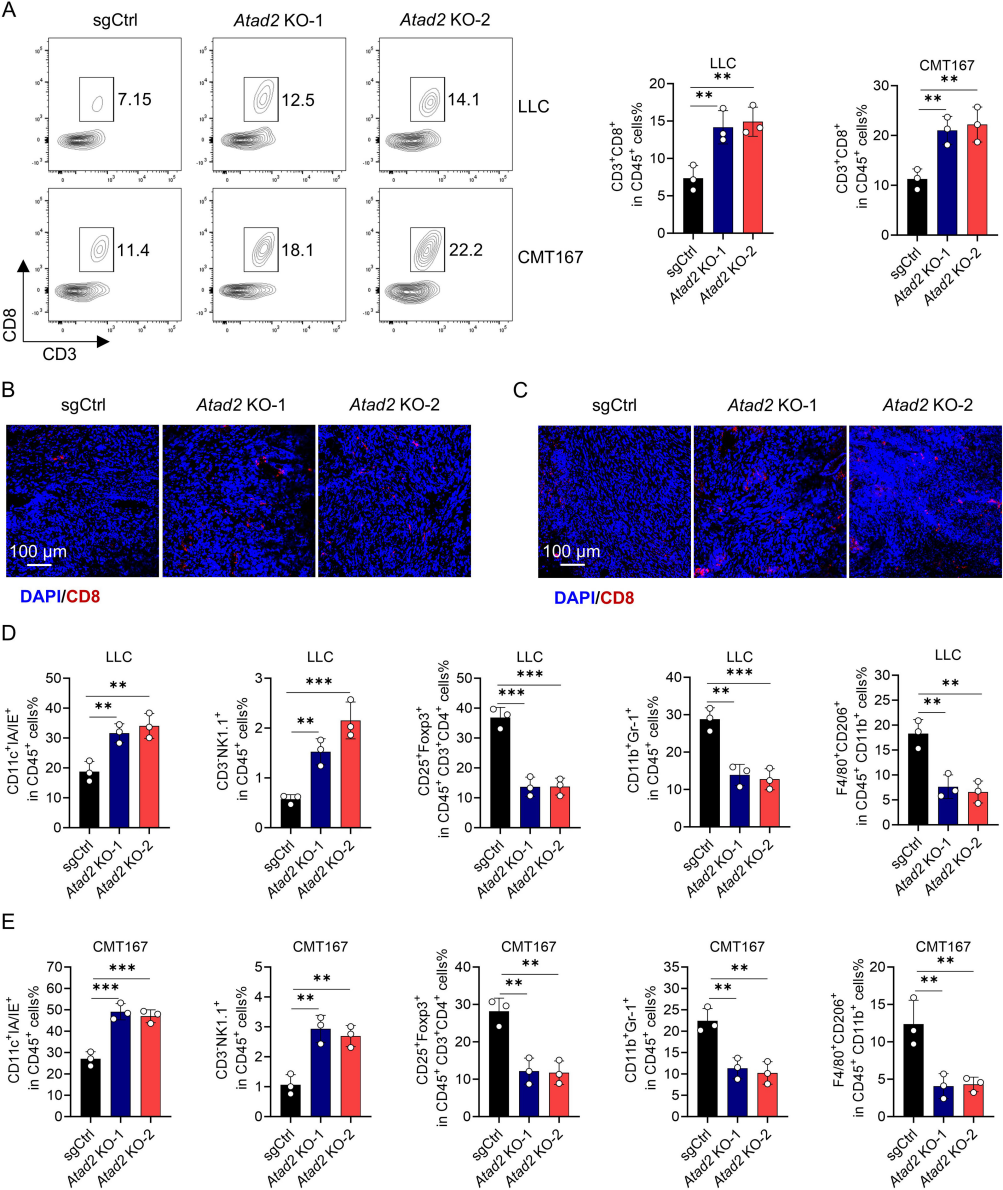
*Atad2* knockout promotes CD8^+^ T cell infiltration and reshapes the intratumoral immune landscape. **(A)** Flow cytometry analysis of tumor-infiltrating CD8^+^ T cells in indicated LLC or CMT167 tumors. (n=3). **(B, C)** Immunofluorescence detection of CD8^+^ T cell infiltration in indicated LLC **(B)** or CMT167 tumor sections **(C)**. (n=3). **(D, E)** Proportions of dendritic cells (CD45^+^CD11c^+^MHC-II^+^), NK cells (CD45^+^CD3^−^ NK1.1^+^), Tregs (CD45^+^CD3^+^CD4^+^CD25^+^Foxp3^+^), MDSCs (CD45^+^CD11b^+^Gr-1^+^), and M2-like TAMs (CD45^+^CD11b^+^F4/80^+^CD206^+^) in LLC **(D)** and CMT167 **(E)** tumors bearing the indicated genotypes. Data are presented as mean ± SD of biological replicates. Representative results from one of three independent experiments are shown. **P < 0.01; ***P < 0.001.

To determine whether *Atad2* loss influences antitumor immunity *in vivo*, we initially assessed the infiltration of CD8^+^ T cells in LLC and CMT167 tumors. Flow cytometric analysis revealed a significant increase in the proportion of CD3^+^CD8^+^ cells among CD45^+^ tumor-infiltrating leukocytes in *Atad2*-deficient tumors compared to controls in both models (**Figure 7A**). In line with this, immunofluorescence staining confirmed a markedly higher accumulation of CD8^+^ T cells within *Atad2*-knockout tumor sections (**Figures 7B, C**).

Given the well-documented pleiotropic effects of LA on diverse immune cell populations, we next investigated whether *Atad2* deletion broadly reshapes the tumor immune microenvironment. Comprehensive flow cytometric profiling of subcutaneous tumors revealed a significant remodeling of the immune landscape. The frequencies of dendritic cells and NK cells were markedly elevated in *Atad2*-deficient tumors relative to controls. Conversely, the proportions of immunosuppressive populations-including Tregs, MDSCs, and M2-like TAMs-were significantly reduced in both tumor models following *Atad2* knockout (**Figures 7D, E**). These broad immune changes are consistent with the known pleiotropic effects of LA on multiple immune lineages, supporting that ATAD2-driven LA production shapes the global immune landscape. Thus, targeting ATAD2 not only reinvigorates CD8^+^ T cells but also alleviates suppression from multiple inhibitory lineages while enhancing the representation of key effector populations.

### *Atad2* deletion augments the efficacy of anti-PD-1 therapy *in vivo*

**Figure 8:**
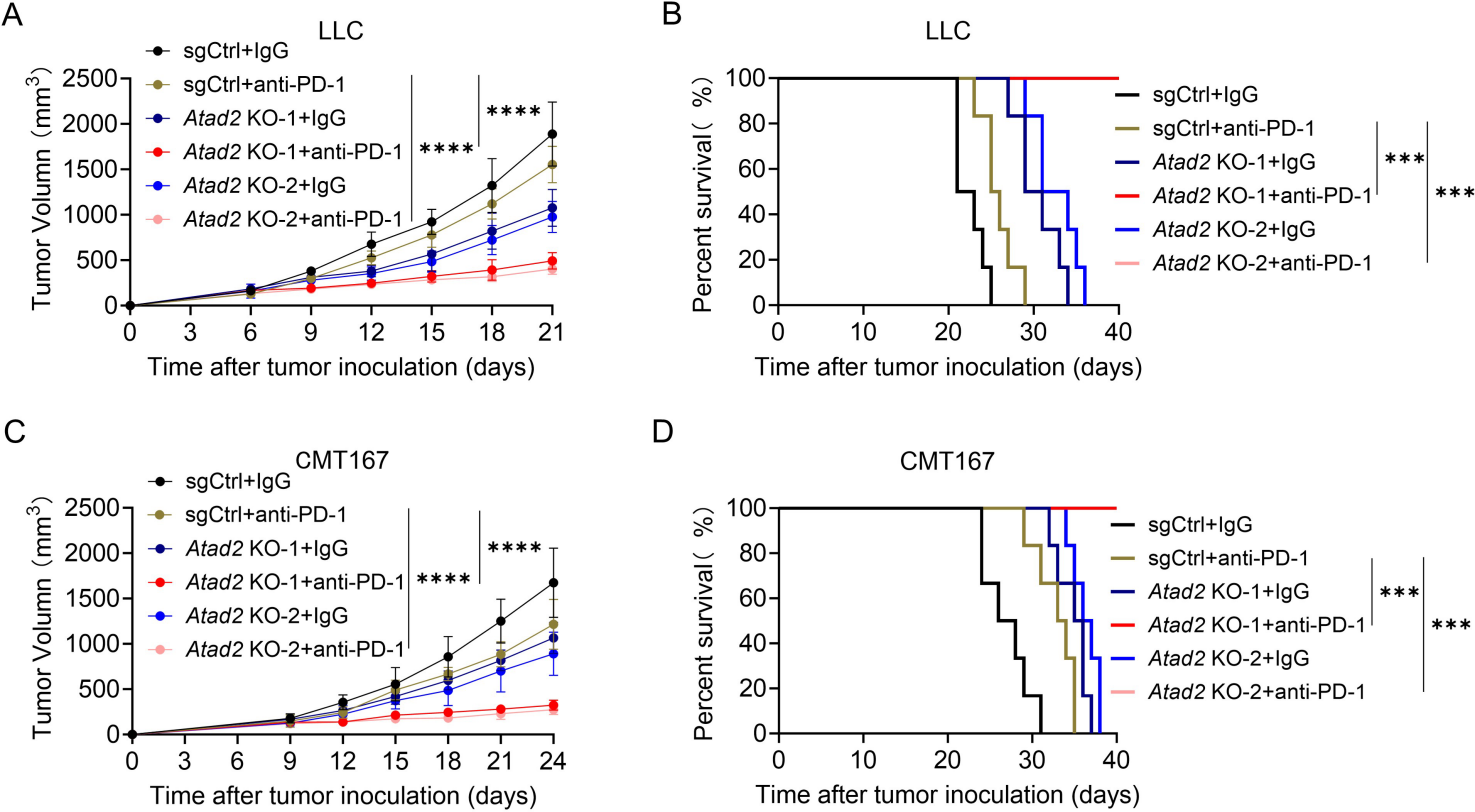
*Atad2* deletion sensitizes tumors to anti-PD-1 immunotherapy *in vivo*. **(A, B)** Tumor growth kinetics **(A)** and overall survival **(B)** of mice implanted with indicated LLC cells with or without anti-PD-1 therapy. (n=6). **(C, D)** Tumor volume progression **(C)** and survival curves **(D)** of mice bearing indicated CMT167 tumors treated with or without anti-PD-1. (n=6). Data are presented as mean ± SD of biological replicates. Representative results from one of three independent experiments are shown. ***P < 0.001; ****P < 0.0001.

Having established that *Atad2* loss remodels the TME towards a more immunostimulatory state, we evaluated its therapeutic potential in combination with ICIs. In the LLC model, genetic ablation of *Atad2* synergized with anti-PD-1 therapy to further restrain tumor progression and significantly prolong survival compared to control groups (**Figures 8A, B**). This enhanced therapeutic effect was recapitulated in the CMT167 model, where *Atad2* deficiency sensitized tumors to PD-1 blockade, leading to reduced tumor burden and extended survival of treated mice (**Figures 8C, D**). Collectively, these findings demonstrate that *Atad2* deficiency not only promotes a favorable intratumoral immune landscape but also potentiates the antitumor efficacy of PD-1 blockade in LUAD models.

## Discussion

In this study, we identify ATAD2 as a pivotal link between epigenetic regulation, metabolic reprogramming, and T cell-based immunotherapy resistance in LUAD. We demonstrate that ATAD2 is markedly upregulated in LUAD and correlates with poor clinical outcomes and diminished CD8^+^ T cell infiltration. Through integrated *in vitro* and *in vivo* analyses, we establish that ATAD2 functions as a transcriptional co-activator. It enhances c-Myc-dependent induction of LDHA, thereby driving LA accumulation within the tumor microenvironment. This LA-enriched milieu suppresses CD8^+^ T cell cytotoxicity and cytokine production while promoting exhaustion. Ultimately, it fosters an immunosuppressive niche permissive to tumor progression and immune evasion.

Although ATAD2 has previously been characterized as an oncogenic epigenetic regulator, our findings extend its role to metabolic-immune modulation (25–27). The ATAD2-LDHA-LA signaling axis identified here provides direct mechanistic insight into how chromatin-associated factors coordinate metabolic remodeling to shape antitumor immunity. Mechanistically, our findings align with and extend the established role of c-Myc as a direct transcriptional activator of LDHA (20, 21). Recent studies have demonstrated that diverse upstream regulators-such as circARHGAP29, METTL5, and NCAPD3-converge on c-Myc to modulate LDHA expression and glycolytic flux in various cancer contexts (22–24). Here we identify ATAD2 as a novel co-activator within this paradigm. Regarding potential upstream signaling, ATAD2 expression has been linked to the PI3K/AKT pathway in LUAD, a cascade known to enhance glycolysis and FDG uptake (14). Moreover, MYC itself can transcriptionally regulate ATAD2, forming a positive feedback loop that reinforces metabolic reprogramming (28). These interconnections suggest that ATAD2 may act as a downstream effector of oncogenic signaling pathways, coupling them to LDHA-mediated immune suppression. Thus, the ATAD2-c-Myc-LDHA axis represents a critical node where epigenetic regulation, metabolic adaptation, and immune evasion converge.

Beyond its effect on CD8^+^ T cells, *Atad2* deficiency profoundly reshaped the broader immune landscape within the TME. We observed significant increases in the infiltration of NK cells and dendritic cells, alongside a marked reduction in immunosuppressive populations including Tregs, MDSCs, and M2-like TAMs. These findings are consistent with the known pleiotropic functions of LA. For instance, LA has been shown to impair NK cell cytotoxicity (29), promote tolerogenic dendritic cell differentiation (30), induce M2-like macrophage polarization (31), and support the stability and function of MDSCs and Tregs within glycolytic tumors (32, 33). These broad immunomodulatory effects position ATAD2 as a high-level regulator of the tumor immune microenvironment. It is therefore an attractive target for combination immunotherapy, as its inhibition may simultaneously alleviate multiple layers of immune suppression.

Our work also highlights the therapeutic relevance of targeting this pathway to restore T cell effector function and enhance immunotherapy efficacy. Potential strategies include developing specific ATAD2 inhibitors to disrupt its transcriptional complex, employing LDHA inhibitors to directly block LA production, or using pharmacological agents that disrupt LA transport (e.g., MCT1 inhibitors) to prevent its accumulation in the TME (34–37). Given the challenges associated with direct LDHA inhibition, ATAD2 may offer an attractive upstream target to indirectly modulate tumor metabolism and reverse immune dysfunction (38, 39). Future studies should prioritize the development of potent ATAD2 inhibitors and evaluate their synergy with ICIs in immunocompetent LUAD models.

Several limitations warrant consideration. First, our ChIP-qPCR data show that *Atad2* knockout reduces c-Myc binding to the LDHA promoter. In addition, c-Myc overexpression restores LDHA expression. These findings support a role for ATAD2 in facilitating c-Myc recruitment. However, direct biochemical evidence-such as Co−IP or ChIP−seq-for an ATAD2-c−Myc interaction or their co−localization at the LDHA promoter in our LUAD models is lacking. Future studies using these approaches are needed to validate this mechanism. Second, while our study identifies LA as a key mediator of ATAD2-driven immune modulation, we cannot exclude the existence of additional, LA-independent pathways. Emerging evidence suggests that ATAD2 may influence antitumor immunity through alternative mechanisms. These include its potential function as a direct immunogenic target presented by HLA molecules (40) and its possible role in promoting tumor angiogenesis, which could indirectly reshape the immune microenvironment (41). Future studies are required to determine whether these pathways operate independently of, or converge with, the ATAD2-LDHA-LA axis identified here. Finally, while our findings are supported by multiple public datasets and extensive *in vitro* and *in vivo* experiments, validation with clinically annotated tumor specimens from immunotherapy-treated LUAD patients would strengthen the translational impact of the ATAD2-LDHA-LA axis. Access to cohorts with matched treatment outcomes and sufficient follow-up data was not available for this study. Future studies should incorporate immunohistochemical analysis of ATAD2 and LDHA expression in both pre- and post-immunotherapy tumor tissues. Correlating this data with patient treatment response and survival will be essential to confirm the clinical significance of our findings.

In conclusion, we identify the ATAD2-LDHA-LA axis as a fundamental metabolic-epigenetic circuit that drives T cell therapy resistance in LUAD by reshaping the tumor microenvironment toward immunosuppression. Our findings not only uncover a previously unrecognized mechanism linking ATAD2 activity to dysfunctional CD8^+^ T cell responses but also position this pathway as a promising therapeutic vulnerability. Targeting ATAD2 or its downstream metabolic effectors can restore T cell function, remodel the tumor immune landscape, and enhance the efficacy of T cell-based immunotherapies in LUAD.

## Data Availability

The original contributions presented in the study are included in the article/supplementary material. Further inquiries can be directed to the corresponding author.
